# Anatomical Variations of Ostiomeatal Complex in CBCT of Patients Seeking Rhinoplasty

**Published:** 2015-03

**Authors:** Leila Khojastepour, Sabah Mirhadi, Seyed Alireza Mesbahi

**Affiliations:** a Dept. of Oral and Maxillofacial Radiology, School of Dentistry, Shiraz University of Medical Science, Shiraz, Iran.; b Dept. of Otolaryngology and Plastic Surgery, Khodadoust Hospital, Shiraz Iran.

**Keywords:** Cone Beam CT, Ostiomeatal Complex, Variations, Mucosal Thickening, Rhinoplasty

## Abstract

**Statement of the Problem:**

Anatomic variation can potentially impact the surgical safety.

**Purpose:**

The purpose of this cross-sectional study was to assess the prevalence of ostiomeatal complex variations based on cone beam computed tomography (CBCT) images of the patients seeking rhinoplasty.

**Materials and Method:**

In this cross-sectional study, CBCT images of 281 patients including 153 female and 128 male with Mean±SD age of 26.97±7.38 were retrieved and analyzed for presence of variations of ostiomeatal complex and mucosal thickening. All CBCT images were acquired by NewTom VGi scanner with 15×15 field of view, as a part of preoperative recording of patients seeking rhinoplasty in an otolaryngology clinic. Chi- square test and Odds ratio were used for statistical analysis of the obtained data and *p*< 0.05 was considered to be statistically significant.

**Results:**

Agger nasi cells which were seen in 93.2% of the cases were the most common anatomic variation. It was followed by Haller cells (68%), concha bullosa (67.3%), uncinate process variations (54.8%), nasal sepal deviation (49.5%) and paradoxical curvature of middle turbinate (10%). Mucosal thickening were detected in 60.7% of the studied cases.

**Conclusion:**

Ostiomeatal complex variations and mucosal thickening are considerably prevalent among the patients seeking rhinoplasty. This study also revealed that CBCT evaluation of paranasal sinuses has comparable result in delineation of the sinonasal anatomy.

## Introduction

Otolaryngologists are interested in radiological assessment of paranasal regional anatomy. [1]Certain anatomical variations of the lateral wall of the nose are very important and possibly contribute to the blockage of the ostiomeatal units, drainage and ventilation, and can thereby, increase the risk of sinus mucosal disease. [2-4] Moreover, anatomic variation could be potentially effective on surgical safety; hence, cross-sectional imaging of bony structures is frequently used as a part of preoperative evaluation. [5] There is not a full agreement about description of ostiomeatal complex. In the present study, the concept developed by Stammberger and Kennedy was adopted. [6] According to Stammberger and Kennedy, ostiomeatal complex is the functional unit of the anterior ethmoid complex and provide final common pathway for drainage and ventilation of the frontal, maxillary and anterior ethmoid sinuses. [6]

Regardless of the controversy about the role of anatomic variations of ostiomeatal complex in inducing rhinosinusitis, being aware of prevalence of these variations might be influential during surgical procedures that involve paranasal sinuses such as Functional Endoscopic Sinus Surgery (FESS) and rhinoplasty. [6-11]

For avoiding dissatisfaction after esthetic rhinoplasty, focus on esthetic improvement of the nasal shape should not sacrifice sinonasal health, [12] and in this regard, preoperative imaging in patients seeking rhinoplasty provide precise evaluation of any medical condition inside the nasal cavity that may lead to unresolved sinonasal problems after the surgery.

Computed tomography (CT) scan is the method of choice for evaluation of paranasal sinuses and the coronal plane is the preferred imaging plane that best displays the ostiomeatal complex. [11]

Moreover, since introducing the first cone beam computed tomography (CBCT) system for dentomaxillofacial imaging in 2001 researches have focused on the feasibility of CBCT in several applications including diagnosis of the problems of nose and paranasal sinuses. Considering sinus scanning protocol, the CBCT systems provide comparable high-contrast resolution and inferior low-contrast resolution relative to those obtained with the multi detector CT scanners )MDCT(. In addition to emitting lower levels of radiation, ﬂat panel CBCT scanners generally have less metal artifact effect in comparison to MDCT. [13]

The relatively low dose and compact design of the equipments made CBCT scanners attractive for diagnosis, surgical planning, and intraoperative applications particularly in the head and neck region. According to the result of recent researches, CBCT images have sufficient quality for visualizing the paranasal sinuses even at lowest radiation exposure. [14-18]Also, a detailed surgical approach for functional nasal defects is easy to be established after a CBCT examination. [19-20]

In line with the advances in imaging technology in the last decade, there is an increased interest toward the details of complex radiological anatomy of the paranasal sinuses and ostiomeatal complex. Several authors investigated the paranasal sinus anatomic variations, particularly the variations of ostiomeatal complex based on conventional MDCT. [1-5, 7-11, 21] ^ ^Recently, Mathew *et al.* reported the prevalence and clinical significance of Haller cells based on CBCT images. [22]

In this study we reviewed the variations of ostiomeatal complex based on CBCT coronal cross sections of the patients who performed CBCT scan for rhinoplasty. 

## Materials and Method

This cross-sectional study evaluated the paranasal sinus CBCT images for presence of anatomical variations of ostiomeatal complex at the Oral Radiology Department of Shiraz University of Medical Sciences. All CBCT images were acquired by NewTom VGi scanner (QR srl; Verona, Italy), with 15×15 field of view, taken as a part of preoperative recording of patients seeking rhinoplasty in an otolaryngology clinic over a 1-year period. Coronal cross sections for each patient were reviewed in NNT workstation by authors, for the following features:

1. The incidence of anatomical variations affecting the ostiomeatal complex including the presence of Concha bullosa (aerated turbinate, most often the middle turbinate), Haller cells (infraorbital ethmoidal air cells), nasal septum deviation, paradoxical middle turbinate (a middle turbinate whose convexity is unusually directed laterally toward the lateral sinus wall), Agger nasi (the most anterior ethmoidal air cells which are located lateral and inferior to the frontal recess), as well as variations in the shape direction and attachment of uncinate process.
2. The incidence of mucosal thickening.

Any alteration of the paranasal sinus anatomy resulting from previous surgery, benign tumors of sinonasal mucosa and facial trauma were considered as exclusion criteria. Data were statistically analyzed using SPSS Software, Version 15 (Chicago; IL, USA). Chi- square test was used for statistical comparison of ostiomeatal anatomic variations between the two genders and between the two sides. 

Odds ratio was used to assess the significance of association between each of the anatomic variations and the presence of mucosal thickening. 

## Results

A total of 281 subjects who met the study criteria including 153 female (54.44%) and 128 male (45.55 %) patients were included in this study. The subjects were 17-52 years old with the Mean±SD age of 26.97±7.38 years. 

**Figure 1:**
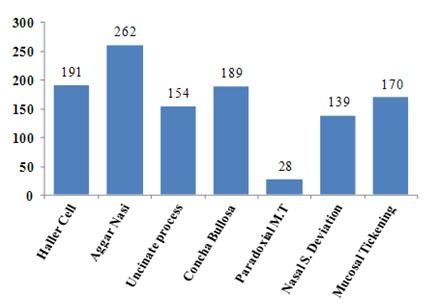
Overall findings of the present study

**Figure 2:**
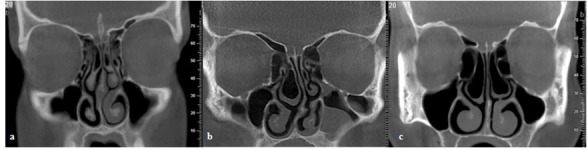
a: Coronal cone bean CT image showing a case with bilateral concha bullosa, variations in superior attachment of both uncinate process (attachment to nasal roof), left uncinate process pneumatization, left paradoxical middle turbinate, Haller cell in the right side and nasal septum deviation  b: Coronal cone bean CT image showing a large right concha bullosa which caused significant nasal septal deviation to the right side.  c: Coronal cone bean CT image showing relatively symmetrical large bilateral concha bullosa and almost straight nasal septum.

Figure 1 shows the overall findings of the present study. A number of ostiomeatal complex anatomical variations found are shown in Figures 2. Nearly all the observed cases (except one) had at least one anatomical variation. In many cases, however, more than one variant existed in the same subject. Being observed in 93.2% of the cases, Agger nasi cells were the most common anatomic variations found; followed by Haller cells (68%), concha bullosa (67.3%), uncinate process variations (54.8%), nasal sepal deviation (49.5%) and paradoxical curvature of middle turbinate (10%), respectively.

Various degrees of mucosal thickening were detected in 60.7% of the studied cases. According to Table 1, the incidence of anatomical variations in this study was more bilaterally. Among the six studied variations, only paradoxical middle turbinate cases occurred more unilaterally. Mucosal thickening was also mostly bilateral; 107 bilateral cases versus 63 case of unilateral mucosal thickening.

As represented in Table 2, regarding unilateral occurrence of anatomical variations, no statistically significant difference was found between the incidence of each variations in the right and left side, except for the concha bullosa which was more common in the right side (*p*= 0.02).

**Table 1:** Incidence of anatomical variations and comparison between unilateral vs. bilateral occurrence of them

**Variations**	**Total No of ** **patients**	**Total Percentage of patients with (%)**	**Unilateral**		**Bilateral**	
			**No**	**%**	**No**	**%**
Haller cell	191	68	65	34.03	126	65.97
AggerNasi	262	93.2	7	2.67	255	97.33
Uncinate Process	154	54.8	73	47.40	81	52.60
CochcaBullosa	189	67.3	75	39.68	114	60.32
Paradoxical M. Turbinate	28	10	24	85.71	4	14.29

**Table 2:** Comparison between Right versus Left side involvement in cases of unilateral occurrence of anatomical variations

**Variations**	**Right**	**Left**	**P value**
Haller cell	33 50.77%	32 49.23%	0.999
Agger Nasi	4 57.2%	3 42.8%	0.999
Uncinate Process	28 38.4%	45 61.6%	0.06
Cochca Bullosa	48 64%	27 36%	0.02
Paradoxical M. Turbinate	8 33.4%	16 66.6%	0.152
Nasal septal deviation	75 54%	64 46%	0.396

*Chi square Test

Table 3 demonstrates that among anatomical variations in this study, only presence of uncinate process variation was statistically significant in men.

**Table 3:** Comparison between incidence of anatomic variations in males and females

**Variations**	**Female**	**Male**	**P value**
Haller cell	108 70.6%	83 64.8%	0.30
AggerNasi	145 94.8%	116 90.6%	0.26
Uncinate Process	71 46.4%	83 64.8%	0.002^*^
Concha bullosa	104 68%	85 66.4%	0.78
Paradoxical M. Turbinate	14 9.2%	14 10.9%	0.61
Nasal septal deviation	68 44.8%	71 55.5%	0.06

*Chi square Test

Prevalence of mucosal thickening was also significantly higher in men than women.

Table 4 shows the relationship between presence of various ostiomeatal complex variations and mucosal thickening. There was significant relation between presence of Haller cells and absence of mucosal thickening (OR=0.51, 95% CI: 0.29-0.87).

There was also significant relation between the presence of Agger nasi cells and absence of mucosal thickening (OR= 0.077, 95%CI: 0.01-0.58).

There was significant relation between the uncinate process variations and presence of mucosal thickening [OR =2.62, 95%CI: 1.60-4.28].

**Table 4:** Relationship between presence of various ostiomeatal complex variations and mucosal thickening

		**Mucosal Thickening**		**Odds Ratio**	**95% Confidence Interval**	P value *
		**0**	**1**			
Haller cell	0	26	64	0.51	0.29 - 0.87	0.007
	1	85	106			
Agar Nasi	0	1	18	0.077	0.01-0.58	0.000
	1	110	152			
Nasal S.D	0	63	79	1.51	0.93-2.45	0.117
	1	48	91			
UP variations	0	66	61	2.62	1.6-4.28	0.000
	1	45	109			
Concha bullosa	0	35	57	0.91	0.55 -1.52	0.6
	1	76	113			
Paradoxical MT	0	98	155	0.73	0.33 - 1.6	0.4
	1	13	15			

*Chi square Test

## Discussion

Regardless of the controversy about the possible role of anatomic variations of paranasal sinus structures in predisposing the patients to recurrent rhinosinusitis, [3, 5, 7-8, 11-12] there is no doubt that these variations should crucially be concerned before and during surgical procedures. These variations are important at least from two different points of view; the first one is their relationship to disrupting drainage and ventilation of paranasal sinuses [14-16] and the second one is their potential impact on operative technique and surgical safety.

Table 5 summarizes the result of our review of articles about the prevalence of anatomic variations of ostiomeatal complex in previous studies. [5, 10-11, 22-23] All of these studies were based on multislice CT except for the study by Mathew *et al.* [22] which evaluated Haller cells prevalence and clinical significance based on CBCT images.

**Table 5:** Summery of some reported prevalence of various ostiomeatal complex variations

**Authors**	**Agger nasi cell**	**Haller's** **cell**	**Uncinate process variations**	**Concha bullosa**	**Nasal septal deviation**	**Paradoxical** **Middle Turbinate**
Lloyd 1990 [23]	3%	2%	16%	14%	NA	17%
Lloyd *et al.* 1991 [24]	14%	15%	21%	24%	NA	18%
Bolger *et al.* 1991 [25]	98.50%	45%	NA	53%	18.8%	26.1%
Scribano *et al.* 1993 [26]	NA	24%	NA	67%	NA	NA
Yousem 1993 [27]	NA	10-45%	NA	34-53%	NA	NA
Wanamaker 1996 [28]	NA	20%	45%	30%	20%	NA
Tonai & Baba 1996 [29]	86.7%	36%	NA	28%	NA	25.3%
Stackpole & Edelstein [30]	NA	34%	NA	NA	NA	NA
Perez-Pinas *et al.* 2000 [31]	Nearly all	3%	4.5%	73%	80%	10%
Zinreich *et al.* 2003 [32]	Nearlly all	10%	3%	36%	21%	NA
Wani *et al.* 2009 [33]	9.33 %	8.66 %	25%	30%	25.33%	9.33%
Alkire *et al.* 2010 [11]	51.8%	70.3%	NA	NA	NA	NA
Mamath *et al. *2010 [10]	50%	17.5%	65%	15%	65%	NA
Fadda *et al. *2012 [5]	24.3%	22.8%	60.5%	49.3%	58.5%	6.4%
Mathew *et al. *2013 [22]	NA	60%	NA	NA	NA	NA
Present study	93.23%	68%	54.8%	67.30%	49.5%	10%

According to Table 5, our findings of paranasal sinuses are generally comparable with those taken by multislice CT. Considering the few studies which compared image quality of CBCT with multislice CT, particularly here in the context of evaluating the sinonasal anatomy, the results of the present study may provide valuable evidence for supporting the adequate feasibility of CBCT images for demonstration of sinonasal bony anatomy.

As displayed in Table 5, the prevalence of Haller cells is remarkably variable, ranging between 2%-70.3%. [5, 10-11, 22-33] In the current study, the prevalence of Haller cell was 68%; which is almost similar to that obtained by Alkire *et al.* [11] and Mathew *et al.* [22] The variability in the reported frequency of Haller cells could be probably associated with inconsistency in definition of Haller cells, mean age of the patients, race, and the CT protocol adopted.

Agger nasi was the most prevalent among the cases investigated in the present study (93.2%); which is comparable with ­­the results of Bolger *et al.* [25] Perez-Pinaset *et al.* [31] Zinreichet *et al.* [32] Much less prevalence for this variation (less than 10%) however were reported by Lloyd [23] and Wani *et al.*, [33] The variability in the reported prevalence of Agger nasi could be related both to its small size and the different definitions assigned to this anatomic variation. [25, 29]

The prevalence of uncinate process variations in this study was 54.8% which correlated with the results of the studies performed by Wanamaker (45%), [28] Mamatha *et al.* (65%); [10] but higher than what were reported by Lloyd (16%), [23] Perez-Pinas *et al.* (4.5%), [31] and Zinreich *et al.* (3%). [32]

The prevalence of concha bullosa in this study was 67.3% and correlated to Bolger *et al.* (53%), [25] Scribano *et al.* (67%), [26] Perez-Pinas *et al.* (73%). [31] It is higher than the reported percentage by Tonai and Baba (28%), [29] Zinreich *et al.* (36%), [32] and Mamatha *et al.* (15%). [10]

It is important to note that the degree of pneumatization could be attributed to racial factors. [29] Badia *et al.* [34] reported ethnic variation in sinonasal anatomy on CT scan of 100 Caucasian and 100 Chinese patients undergoing ESS. More recently, Rashid Al-Abri *et al.* [35] evaluated the clinically significant anatomical variations of the paranasal sinuses in the Omani population and found an ethnical difference in the prevalence of anatomical variations. [35]

The prevalence of paradoxical middle turbinate in this study was 10% which is in line with the study performed by Perez-Pinas *et al.* (10%) [31] and Lloyd (17%). [23, 31]

As shown in Table 5, the prevalence of nasal septal deviation also is quite variable in this study (49.5%).All detected Haller cell in the study carried out by Wani *et al.* [33] were unilateral, while Fadda *et al.* [5]reported equal unilateral and bilateral Haller cells. In this study, however, Haller cell were unilateral in 34.03% and bilateral in 65.97% of the cases.

Nearly all Agger nasi cells detected in the current study were bilateral (97.33% vs. 2.67%). This variation was also more bilateral among the cases investigated by Fadda *et al.*; [5] whereas according to the results found by Wani *et al.*, [33] Agger nasi had almost similar bilateral and unilateral presentation (4% vs. 5.33%). In our cases concha bullosa were mostly presented bilaterally (60.32% VS 39.68%) which is in contrast with Wani *et al.* [33] and Fadda *et al.* [5]studies in which concha bullosa were detected mostly unilaterally.

In this study, unilateral paradoxical curvature of middle turbinate (85.71%) were detected to be more than bilateral ones; which was in accordance with the studies by Wani *et al.* [33] and Fadda *et al.* Bilateral and unilateral occurrence of uncinate process variations was almost similar in our cases (47.40% vs. 52.60%) while in the studies by both Wani *et al.* [33] and Fadda *et al.* [5]unilateral occurrence of uncinate process were more common.

Picavet *et al.* [12]performed nasal endoscopy on 269 patients seeking rhinoplasty to evaluate anatomic and/or mucosal disease. They reported structural pathology in 62% of rhinoplasty patients while nasal septal deviation with prevalence of 54% was the most frequent problem. However, it was not in agreement with the findings of the current study in which nasal septal deviation was not the most frequent structural finding.

In addition, we found mucosal thickening in 60.7% of cases while Picavet *et al.* [12] found mucosal disease only in 28% of rhinoplasty patients. These differences could be partially related to higher precision of different diagnostic modalities which were used.

Based on Mathew *et al.*, [22] there was no significant relation between the presence and size of Haller cell and maxillary sinusitis. Nevertheless, the result of some studies revealed a relation between the presence of Haller cells and chronic rhinosinusitis. [5, 8, 26, 30] It is also true about other ostiomeatal variations such as Agger nasi and uncinate process variations. [5, 8-9] According to the results of the present study the prevalence of Haller cells, Agger nasi cells and uncinate process variations were more common in those cases who had mucosal thickening.

Considering odds ratio, uncinate process variations predisposed the cases to mucosal thickening which is in accordance with the previous studies. [3, 5, 8] Additionally, odds ratio suggest protective role for Haller cells and /or Agger nasi in developing mucosal thickening, which is in contrast with previous studies. This difference could be explained by the fact that participants in the present study were cases who seek rhinoplasty rather than chronic rhinosinusitis cases.

## Conclusion

Ostiomeatal complex variations and mucosal thickening have considerable prevalence among patients seeking rhinoplasty. To reduce the possible complications of the surgery and to achieve optimum satisfactory results, these structural and mucosal alterations could deliberately be evaluated by CBCT with relatively lower radiation exposure. 
